# Hybridization within *Saccharomyces* Genus Results in Homoeostasis and Phenotypic Novelty in Winemaking Conditions

**DOI:** 10.1371/journal.pone.0123834

**Published:** 2015-05-06

**Authors:** Telma da Silva, Warren Albertin, Christine Dillmann, Marina Bely, Stéphane la Guerche, Christophe Giraud, Sylvie Huet, Delphine Sicard, Isabelle Masneuf-Pomarede, Dominique de Vienne, Philippe Marullo

**Affiliations:** 1 INRA, UMR 0320 / UMR 8120 Génétique Végétale, Gif-sur-Yvette, France; 2 ENSCBP—Bordeaux INP, Pessac, France; 3 Université de Bordeaux, ISVV, EA 4577, Unité de recherche Œnologie, Villenave d'Ornon, France; 4 Université Paris-Sud, UMR 0320 / UMR 8120 Génétique Végétale, Gif-sur-Yvette, France; 5 SARCO, Bordeaux, France; 6 CMAP Ecole Polytechnique, Palaiseau, France; 7 INRA, MIA, Jouy-en-Josas, France; 8 Bordeaux Sciences Agro, Gradignan, France; 9 Biolaffort, Bordeaux, France; University of Strasbourg, FRANCE

## Abstract

Despite its biotechnological interest, hybridization, which can result in hybrid vigor, has not commonly been studied or exploited in the yeast genus. From a diallel design including 55 intra- and interspecific hybrids between *Saccharomyces cerevisiae* and *S*. *uvarum* grown at two temperatures in enological conditions, we analyzed as many as 35 fermentation traits with original statistical and modeling tools. We first showed that, depending on the types of trait – kinetics parameters, life-history traits, enological parameters and aromas –, the sources of variation (strain, temperature and strain * temperature effects) differed in a large extent. Then we compared globally three groups of hybrids and their parents at two growth temperatures: intraspecific hybrids *S*. cerevisiae * S. *cerevisiae*, intraspecific hybrids *S*. uvarum * S. *uvarum* and interspecific hybrids *S*. cerevisiae * S. *uvarum*. We found that hybridization could generate multi-trait phenotypes with improved oenological performances and better homeostasis with respect to temperature. These results could explain why interspecific hybridization is so common in natural and domesticated yeast, and open the way to applications for wine-making.

## Introduction

Plant hybrids commonly harbor non-additive inheritance for polygenic traits, with phenotypic values usually different from the mean parental values. These “monsters” produced by hybridization [1] have evolutionary implications [2, 3] and are extensively exploited for producing improved crops. For instance in maize, F1 hybrids between homozygous lines show heterosis of 100 to 400% for grain yield [4, 5], and many other complex traits, such as height, leaf area, grain size, germination rate, root growth and root nitrogen uptake, also display heterosis [6, 7].

Hybridization affects not only the phenotypic values, but also their stability over environmental changes. Homeostasis, canalization or robustness— the term depends on the biological field [8–11] allows the organisms to buffer the effects of external perturbations through metabolic, physiological or developmental adjustments, and thus to maintain fitness in diverse habitats. Homeostasis is usually higher in intra- or interspecific hybrids than in their parents, as shown for instance for yield, tolerance to soil acidity and to soil moisture stress in maize [12] or morphometric traits in mice [13].

Consequences of hybridization have been studied in a large range of wild as well as domesticated species, but have scarcely been studied and exploited in industrial eukaryotic micro-organisms such as yeast. Recently some authors investigated the heterosis phenomenon within natural and domesticated strains of *Saccharomyces cerevisiae* [14–17], but these studies were mainly focused on cell growth in laboratory conditions.

In the *Saccharomyces sensu stricto* clade, yeast species showed a severe reproductive isolation (less than 1% of viable spores)[18]. However, the prezygotic barrier can be easily bypassed leading to viable interspecific hybrids [19]. Numerous interspecific hybrids between *S*. *cerevisiae* and psychrophilic species *S*. *uvarum* or *S*. *kudriavzevii* have been isolated in wine and natural environment [20–28]. These natural hybrids have technological properties differing from those of their respective “parental” species, with sometimes better robustness [29–32]. Moreover some wine starters empirically selected proved to be interspecific hybrids [25, 33, 34], promoting the idea that interspecific hybridization is a good way for obtaining valuable strains for wine fermentation. However in the previous works the parental strains of the hybrids were not known, so it was not possible to state definitely that interspecific hybridization conferred novel phenotypes and possibly better homeostasis. Moreover, since their genesis, these natural hybrids may have undergone genomic modifications that can drastically affect their phenotype such gross chromosomal rearrangement [35, 36], loss of heterozygosity [23, 37], particular mitotypes [38], aneuploidies [37] and introgressions [39].

In order to assess rigorously the phenotypic impact of intra- and interspecific hybridization, the hybrids must be compared to their parental strains. As previously reviewed [19, 40] various laboratories have produced such hybrids between *Saccharomyces* species [27, 41–43]. However, only a few interspecific hybrids were compared to their parents, and for quite a small number of traits [44–46]. As the *Saccharomyces* strains harbor huge genetic and phenotypic diversity [47, 48], the behavior of few hybrids is not sufficient to have an overall view on the effects of hybridization.

In this work we examined the extent to which hybridization within and between *Saccharomyces* species modified a large series of traits measured during and at the end of fermentation at two temperatures, with particular attention to homeostasis. We focused on *S*. *cerevisiae* and *S*. *uvarum* (formerly *S*. *bayanus* var. *uvarum*) [28, 49, 50], two related species naturally associated with wine fermentations [51–53]. *S*. *cerevisiae* is the main yeast able to achieve grape must fermentation, but *S*. *uvarum* can display similar fermentation performance, particularly at low temperature [40, 54–56]. Although these sister species share large synteny [57, 58], they differ for several technological traits. First, *S*. *cerevisiae* has a higher resistance to high temperature stress (up to 37°C) [30] while *S*. *uvarum* is more tolerant to low temperatures [59]. Second, *S*. *uvarum* exhibits a specific aromatic profile by producing higher amounts of phenyl-2-ethanol and phenyl-2-ethanol acetate than *S*. *cerevisiae* strains [31, 41, 44]. Finally, although *S*. *uvarum* harbors a high ethanol resistance (up to 15% [41]), it is less resistant than *S*. *cerevisiae* [60]. Several natural hybrids between these two species have been described [26, 30, 61], and the possibility to produce synthetic inter-specific hybrids [62] established *S*. *cerevisiae* and *S*. *uvarum* as model systems for hybridization studies.

Measuring an unprecedented number of traits, we investigated the physiological and technological properties of a collection of four *S*. *uvarum* and seven *S*. *cerevisiae* parental strains and their 55 possible hybrids, namely 27 intraspecific hybrids and 28 inter-specific hybrids, under winemaking conditions at two temperatures. We analyzed the sources of phenotypic variation— genetic and/or environmental —for various categories of traits (fermentation kinetics, life-history, wine composition and organoleptic quality), we compared the intra- and interspecific hybrids and assessed the extent to which hybridization increased homeostasis at a multi-trait level.

## Materials and Methods

### Parental strains and culture conditions

**Fig 1:**
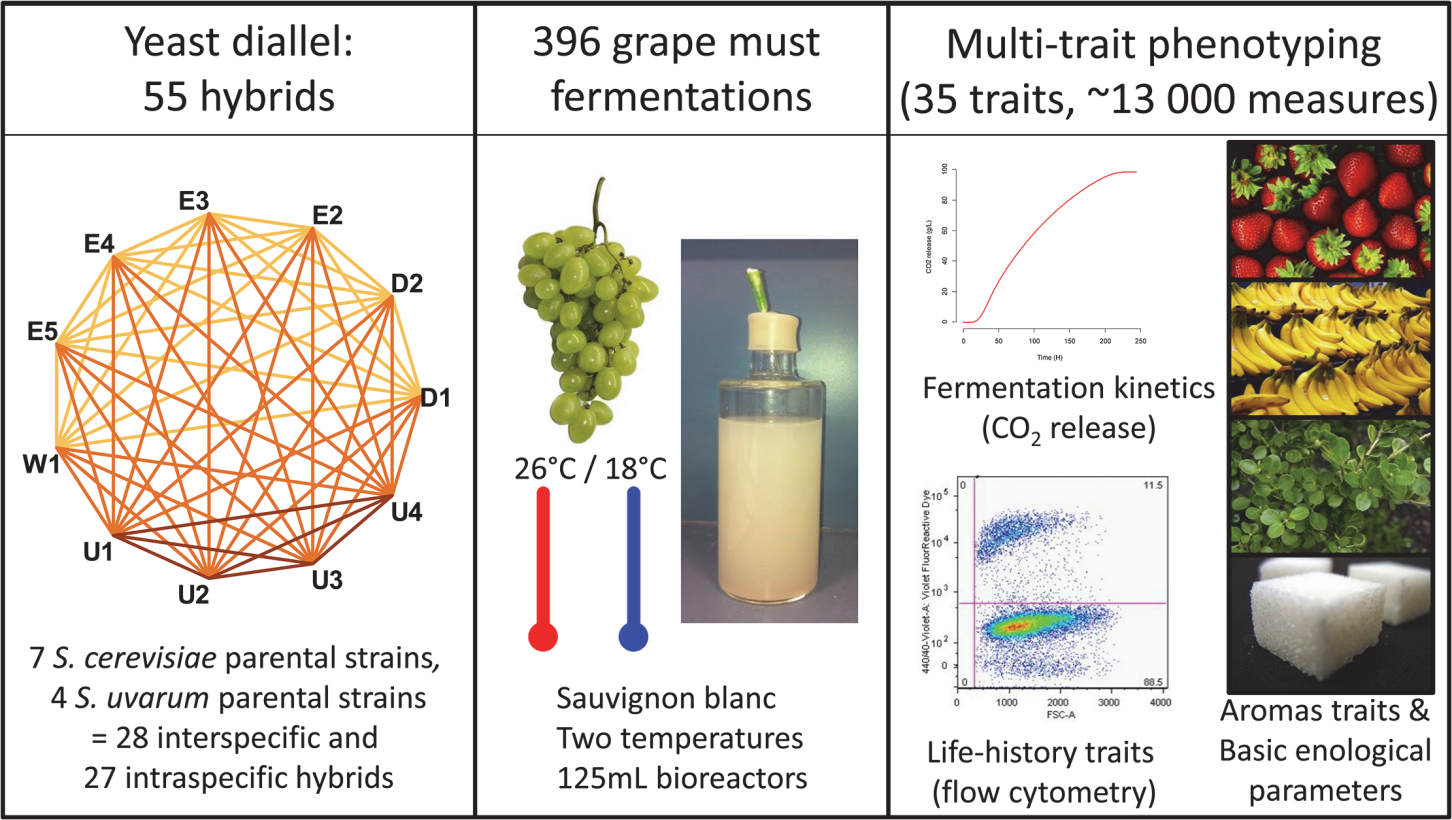
Experimental design. Fully homozygous diploid strains were used as parental strains. W1, D1, D2, E2, E3, E4 and E5 are *S*. *cerevisiae* strains. U1, U2, U3 and U4 are *S*. *uvarum* strains. Fermentations were carried out in Sauvignon blanc grape juice and run at two temperatures 18°C and 26°C in triplicate for a total of 396 experiments. Thirty-five traits were collected and grouped into four classes (Fermentation Kinetics Traits, Life History Traits, Basic Enological Parameters and Aromatic Traits). Fruits, plant and sugar images were reprinted from CC BY licenses, the copyright holders being Steve Hopson, 2006 (bananas); Uwe Hermann, 2007 (sugar cubes); Sharon Mollerus, 2007 (strawberries); Sten Porse, 2005 (boxwood); Lebensmittelfotos, 2013 (grapes).

The starting genetic material of the experimental design were seven *S*. *cerevisiae* strains and four *S*. *uvarum* strains, associated to various food-processes (enology, brewery, cider fermentation and distillery) or isolated from natural environment (oak exudates) (Table 1). These strains could not be used as such as parents of a diallel design because they were suspected to be heterozygous at many loci. Monosporic clones were isolated by tetrad dissection using a micromanipulator (Singer MSM Manual; Singer Instrument, Somerset, United Kingdom). All original strains but Alcotec 24 were homothallic (*HO*/*HO*), therefore fully homozygous diploid strains were spontaneously obtained by fusion of opposite mating type cells. For A24 (*ho/ho*), one isolated haploid meiospore was diploidized via transient expression of the HO endonuclease [63]. These strains, called W1, D1, D2, E2, E3, E4 and E5 for *S*. *cerevisiae* and U1, U2, U3 and U4 for *S*. *uvarum*, were used as the parental strains for the construction of a half diallel design (Fig 1).

**Table 1:** Yeast strains used in this study.

Strain	Genotype	Ploidy	Collection/supplier	Origin	Reference
*Original strains*					
YSP128	*HO/HO* (*S*. *cerevisiae*)	diploid	SGRP	Forest Oak exudate, Pennsylvania, USA	[64]
Alcotec 24	*ho/ho* (*S*. *cerevisiae*)	diploid	Hambleton Bard	Distillery, UK	[65]
CLIB-294	*HO/HO* (*S*. *cerevisiae*)	diploid	CIRM-Levures	Distillery, Cognac, France	[65]
VL1	*HO/HO* (*S*. *cerevisiae*)	diploid	Laffort Œnologie	Enology, Bordeaux, France	[66]
F10	*HO/HO* (*S*. *cerevisiae*)	diploid	Laffort Œnologie	Enology, Bordeaux, France	[67]
VL3c	*HO/HO* (*S*. *cerevisiae*)	diploid	Laffort Œnologie	Enology, Bordeaux, France	[68]
BO213	*HO/HO* (*S*. *cerevisiae*)	diploid	Laffort Œnologie	Enology, Bordeaux, France	[66]
PM12	*HO/HO* (*S*. *uvarum*)	diploid	EA OENOLOGY	Grape must fermentation, Jurançon, France	[69]
PJP3	*HO/HO* (*S*. *uvarum*)	diploid	EA OENOLOGY	Grape must fermentation, Sancerre, France	[69]
Br6.2	*HO/HO* (*S*. *uvarum*)	diploid	ADRIA Normandie	Cider fermentation, Normandie, France	[62]
RC4-15	*HO/HO* (*S*. *uvarum*)	diploid	EA OENOLOGY	Grape must fermentation, Alsace, France	[69]
*Homozygous diploid parental strains*					
W1	Derived from YSP128, HO/HO	diploid	EA OENOLOGY		[54]
D2	Derived from Alcotec24, ho/ho	diploid	EA OENOLOGY		[65]
D1	Derived from CLIB-294, HO/HO	diploid	EA OENOLOGY		[65]
E3	Derived from VL1, HO/HO	diploid	EA OENOLOGY		[65]
E4	Derived from F10, HO/HO	diploid	EA OENOLOGY		[65]
E5	Derived from VL3c, HO/HO	diploid	EA OENOLOGY		[54]
E2	Derived from BO213, HO/HO	diploid	EA OENOLOGY		[67]
U1	Derived from PM12, HO/HO	diploid	EA OENOLOGY		[54]
U2	Derived from PJP3, HO/HO	diploid	EA OENOLOGY		[54]
U3	Derived from Br6.2, HO/HO	diploid	EA OENOLOGY		[54]
U4	Derived from RC4-15, HO/HO	diploid	EA OENOLOGY		this work
*Monosporic clones used for crosses*					
D1-HYG-1A	*ho*::*hygR*, *MATa*	haploid	EA OENOLOGY		this work
D1-HYG-4C	*ho*::*hygR*, *MATalpha*	haploid	EA OENOLOGY		this work
D2-3A-HYG	*ho*::*hygR*, *MATalpha*	haploid	EA OENOLOGY		[62]
E2-KAN-4A	*ho*::*kanR*, *MATalpha*	haploid	EA OENOLOGY		this work
E2-KAN-4D	*ho*::*kanR*, *MATa*	haploid	EA OENOLOGY		this work
E3-NAT-1C	*ho*::*natR*, *MATa*	haploid	EA OENOLOGY		this work
E3-NAT-2C	*ho*::*natR*, *MATalpha*	haploid	EA OENOLOGY		this work
E3-KAN-1B	*ho*::*kanR*, *MATa*	haploid	EA OENOLOGY		this work
E4-NAT-3A	*ho*::*natR*, *MATalpha*	haploid	EA OENOLOGY		this work
E4-NAT-3B	*ho*::*natR*, *MATa*	haploid	EA OENOLOGY		this work
E5-KAN-1C	*ho*::*kanR*, *MATa*	haploid	EA OENOLOGY		this work
E5-HYG-5B	*ho*::*hygR*, *MATalpha*	haploid	EA OENOLOGY		this work
E5-HYG-5D	*ho*::*hygR*, *MATa*	haploid	EA OENOLOGY		this work
W1-NAT-1B	*ho*::*natR*, *MATa*	haploid	EA OENOLOGY		[62]
W1-NAT-1C	*ho*::*natR*, *MATalpha*	haploid	EA OENOLOGY		this work
U1-KAN-4A	*Suho*::*kanR*, *MATalpha*	haploid	EA OENOLOGY		this work
U1-KAN-5D	*Suho*::*kanR*, *MATa*	haploid	EA OENOLOGY		this work
U2-KAN-2A	*Suho*::*kanR*, *MATa*	haploid	EA OENOLOGY		this work
U2-KAN-3B	*Suho*::*kanR*, *MATalpha*	haploid	EA OENOLOGY		[62]
U3-KAN-3A	*Suho*::*kanR*, *MATa*	haploid	EA OENOLOGY		[62]
U3-KAN-3B	*Suho*::*kanR*, *MATalpha*	haploid	EA OENOLOGY		this work
U4-KAN-2C	*Suho*::*kanR*, *MATa*	haploid	EA OENOLOGY		this work
U4-KAN-2B	*Suho*::*kanR*, *MATalpha*	haploid	EA OENOLOGY		this work
*Hybrids of the diallel design*					
DD12	D1-HYG-1A * D2-3A-HYG	diploid	EA OENOLOGY		this work
DE12	D1-HYG-1A * E2-KAN-4A	diploid	EA OENOLOGY		this work
DE13	D1-HYG-1A * E3-NAT-2C	diploid	EA OENOLOGY		this work
DE14	D1-HYG-1A * E4-NAT-3A	diploid	EA OENOLOGY		this work
DE15	D1-HYG-4C * E5-KAN-1C	diploid	EA OENOLOGY		this work
DE22	D2-3A-HYG * E2-KAN-4D	diploid	EA OENOLOGY		this work
DE23	D2-3A-HYG * E3-NAT-1C	diploid	EA OENOLOGY		this work
DE24	D2-3A-HYG * E4-NAT-3B	diploid	EA OENOLOGY		this work
DE25	D2-3A-HYG * E5-KAN-1C	diploid	EA OENOLOGY		this work
DU11	D1-HYG-1A * U1-KAN-4A	diploid	EA OENOLOGY		this work
DU12	D1-HYG-1A * U2-KAN-3B	diploid	EA OENOLOGY		this work
DU13	D1-HYG-1A * U3-KAN-3B	diploid	EA OENOLOGY		this work
DU14	D1-HYG-1A * U4-KAN-2B	diploid	EA OENOLOGY		this work
DU21	D2-3A-HYG * U1-KAN-5D	diploid	EA OENOLOGY		this work
DU22	D2-3A-HYG * U2-KAN-2A	diploid	EA OENOLOGY		this work
DU23	D2-3A-HYG * U3-KAN-3A	diploid	EA OENOLOGY		this work
DU24	D2-3A-HYG * U4-KAN-2C	diploid	EA OENOLOGY		this work
DW11	D1-HYG-1A * W1-NAT-1C	diploid	EA OENOLOGY		this work
DW21	D2-3A-HYG * W1-NAT-1B	diploid	EA OENOLOGY		this work
EE23	E2-KAN-4A * E3-NAT-1C	diploid	EA OENOLOGY		this work
EE24	E2-KAN-4D * E4-NAT-3A	diploid	EA OENOLOGY		this work
EE25	E2-KAN-4A * E5-HYG-5D	diploid	EA OENOLOGY		this work
EE34	E3-KAN-1B * E4-NAT-3A	diploid	EA OENOLOGY		this work
EE35	E3-NAT-2C * E5-KAN-1C	diploid	EA OENOLOGY		this work
EE45	E4-NAT-3A * E5-KAN-1C	diploid	EA OENOLOGY		this work
EU21	E2-KAN-4A * U1-KAN-5D	diploid	EA OENOLOGY		this work
EU22	E2-KAN-4A * U2-KAN-2A	diploid	EA OENOLOGY		this work
EU23	E2-KAN-4A * U3-KAN-3A	diploid	EA OENOLOGY		this work
EU24	E2-KAN-4A * U4-KAN-2C	diploid	EA OENOLOGY		this work
EU31	E3-NAT-1C * U1-KAN-4A	diploid	EA OENOLOGY		this work
EU32	E3-NAT-1C * U2-KAN-3B	diploid	EA OENOLOGY		this work
EU33	E3-NAT-1C * U3-KAN-3B	diploid	EA OENOLOGY		this work
EU34	E3-NAT-1C * U4-KAN-2B	diploid	EA OENOLOGY		this work
EU41	E4-NAT-3B * U1-KAN-4A	diploid	EA OENOLOGY		this work
EU42	E4-NAT-3B * U2-KAN-3B	diploid	EA OENOLOGY		this work
EU43	E4-NAT-3B * U3-KAN-3B	diploid	EA OENOLOGY		this work
EU44	E4-NAT-3B * U4-KAN-2B	diploid	EA OENOLOGY		this work
EU51	E5-HYG-5D * U1-KAN-4A	diploid	EA OENOLOGY		this work
EU52	E5-HYG-5D * U2-KAN-3B	diploid	EA OENOLOGY		this work
EU53	E5-HYG-5D * U3-KAN-3B	diploid	EA OENOLOGY		this work
EU54	E5-HYG-5D * U4-KAN-2B	diploid	EA OENOLOGY		this work
EW21	E2-KAN-4A * W1-NAT-1B	diploid	EA OENOLOGY		this work
EW31	E3-KAN-1B * W1-NAT-1C	diploid	EA OENOLOGY		this work
EW41	E4-NAT-3A * W1-NAT-1B	diploid	EA OENOLOGY		this work
EW51	E5-HYG-5B * W1-NAT-1B	diploid	EA OENOLOGY		this work
UU12	U1-KAN-4A * U2-KAN-2A	diploid	EA OENOLOGY		this work
UU13	U1-KAN-4A * U3-KAN-3A	diploid	EA OENOLOGY		this work
UU14	U1-KAN-4A * U4-KAN-2C	diploid	EA OENOLOGY		this work
UU23	U2-KAN-3B * U3-KAN-3A	diploid	EA OENOLOGY		this work
UU24	U2-KAN-2A * U4-KAN-2B	diploid	EA OENOLOGY		this work
UU34	U3-KAN-3B * U4-KAN-2C	diploid	EA OENOLOGY		this work
WU11	W1-NAT-1B * U1-KAN-4A	diploid	EA OENOLOGY		this work
WU12	W1-NAT-1B * U2-KAN-3B	diploid	EA OENOLOGY		this work
WU13	W1-NAT-1B * U3-KAN-3B	diploid	EA OENOLOGY		this work
WU14	W1-NAT-1B * U4-KAN-2B	diploid	EA OENOLOGY		this work

All strains were grown at 24°C in YPD medium containing 1% yeast extract (Difco Laboratories, Detroit, MI), 1% Bacto peptone (Difco), and 6% glucose, supplemented or not with 2% agar. When necessary, antibiotics were added at the following concentrations: 100 μg/mL for G418 (Sigma, l’Isle d’Albeau France) or nourseothricin (Werner Bioagent, Jena, Germany), and 300 μg/mL for hygromycin B (Sigma).

### Construction of the diallel design

In order to produce interspecific hybrids, the eleven diploid parental strains were transformed with a cassette containing the *HO* allele disrupted by a gene of resistance to either G418 (*ho*::*KanR*), hygromycin B (*ho*::*HygR*) or nourseothricin (*ho*::*NatR*) as previously described [62]. After transformation, monosporic clones were isolated, and the mating-type (*MATa* or *MATα*) of antibiotic-resistant clones was determined using testers of known mating-type. Strain transformation allowed (i) conversion to heterothallism for the homothallic strains (all but D2, see Table 1) and (ii) antibiotic resistance allowing easy hybrid selection.

For each hybrid construction, parental strains of opposite mating type were put in contact for 2 to 6 hours in YPD medium at room temperature, and then plated on YPD-agar containing the appropriate antibiotics. The 55 possible hybrids from the 11 parental strains, namely 21 *S*. *cerevisiae* intraspecific hybrids, 6 *S*. *uvarum* intraspecific hybrids and 28 interspecific hybrids, were obtained. For each cross, a few independent colonies were collected. After recurrent cultures on YPD-agar corresponding to ~ 80 generations, the nuclear chromosomal stability of the hybrids was controlled by pulsed field electrophoresis (CHEF-DRIII, Biorad, CA) as well as homoplasmy (only one parental mitochondrial genome) as detailed in Albertin *et al*. [62].

### Yeast strain characterization

In order to discriminate rapidly the hybrids and parental strains, we used two polymorphic microsatellites specific to *S*. *cerevisiae* (Sc-YFR038, Sc-YML091) [70] and two specific to *S*. *uvarum* (locus 4 and 9) [69]. These four markers were amplified in a multiplex PCR reaction with the labeled primers (S1 Table). The PCR was carried out in a final volume of 8 μL using the following program: 95°C for 5 min for initial denaturation step; 95°C for 30 s, 55°C for 90 s and 72°C for 60 s repeated 35 times; a final elongation step of 30 min at 60°C. The PCR products were analyzed on an ABI3730 apparatus (Applied Biosystem, Villebon-sur-Yvette, France) by the genotyping facilities of Bordeaux University. Microsatellite lengths were analyzed using the Peak Scanner tool (Applied Biosystem, Villebon-sur-Yvette, France)

### Alcoholic fermentation experiments

#### Experimental design

The 66 strains (11 parental and 55 hybrids) were grown in three replicates at two temperatures, 26°C and 18°C. The 396 fermentations (66 strains x 2 temperatures x 3 replicates) were performed following a randomized experimental design. The design was implemented considering a block as two sets of 27 fermentations (26 fermentations and a control without yeast to check for contamination), one carried out at 26°C and the other at 18°C. The distribution of the strains within the blocks was randomized to minimize the residual variance of the estimators of the *Strain* and *Temperature* effects.

#### Grape must and fermentation conditions

White grape must was obtained from *Sauvignon blanc* grapes, harvested in vineyards in Bordeaux area (2009 vintage). This grape juice was obtained by Ducourt Vignoble 18 Le Hourc, 33760 Ladaux that gives the autorization to use this material for the study. The harvest was realized by the owner of the field and did not involve endangered or protected species.Tartaric acid precipitation was stabilized and turbidity was adjusted to 100 NTU (Nephelometric Turbidity Unit) before storage at – 20°C. Grape juice had a sugar concentration of 189 g.L^–1^, a pH of 3.3 and an assimilable nitrogen content of 242 mg N.L^–1^. The indigenous yeast population was estimated by YPD-plate counting after must thawing and was consistently lower than 20 CFU (Colony-Forming Unit) *per* mL.

Yeast pre-cultures (20 mL) were run in half-diluted must filtered through a 0.45 μm nitrate-cellulose membrane, during 24 h, at 24°C with orbital agitation (150 rpm). Cell concentration was quantified using a flow cytometer (see below) and grape must was inoculated at 10^6^ viable cells *per* mL. Fermentations were run in 125 mL glass-reactors, locked to maintain anaerobiosis, with permanent stirring (300 rpm) at 18°C or 26°C. Yeast strain implantation in grape must was checked when the stationary phase was reached (40% of alcoholic fermentation). The DNA of fermenting yeast was extracted using FTA clone saver cards (Whatman, France), and strain identity was controlled by microsatellite analysis.

#### Flow cytometry analysis

The monitoring of population growth, cell size and viability was performed using a cytometer FC500 MPL (Beckman Coulter, Villepinte, France). Collected samples were filtered before flow cytometry analysis with 10 μm disposable filters, CellTric (Partec, Münster, Germany). Samples were diluted with McIlvaine buffer pH 4 (0.1 M citric acid, 0.2 M sodium phosphate dibasic) added with propidium iodide (0.3% v/v) in order to stain dead cells, and dilution was adapted to reach a flow rate lower than 2500 particules/sec. Fluorescent beads (Cell Counter, Beckman Coulter) were used to normalize the quantification of cellular concentration.

### Multi-trait phenotyping in winemaking conditions

For each alcoholic fermentation, four sets of experimental data were obtained: fermentation kinetics parameters (FK), life-history traits (LHT), basic enological parameters (BEP) and aromatic traits (AT).

#### Fermentation kinetics parameters (FK)

The amount of CO_2_ released was monitored daily by the weight loss of the bioreactors.

The amount of CO_2_ released (*Y*_*it*_) for the fermentation *i* at time *t* was modeled by a Weibull function *f* as described in S1 Supporting Information. Seven kinetics parameters were computed from the model (S1 Fig):

- *t-lag* (h) = ${\hat{t}}_{0}$, the fermentation lag-phase that is the time between inoculation and the beginning of CO_2_ release (when the CO_2_ production rate was higher than 0.05 g.L^–1^.h^–1^);
- *t-V*_max_ (h), the time to reach the inflexion point, out of the fermentation lag phase;
- *t-45* (h), the fermentation time at which 45 g.L^–1^ of CO_2_ was released, out of the fermentation lag phase;
- *t-75* (h), the fermentation time at which 75 g.L^–1^ of CO_2_ was released, out of the fermentation lag phase;
- *AFtime* (Alcoholic Fermentation time, h), the time between *t-lag* and the time at which the CO_2_ emission rate became less than, or equal to, 0.05 g.L^–1^.h^–1^;
- *V*_max_ (g.L^–1^.h^–1^), the value of the first derivative of the Weibull function *f*, at *t-V*max (h), *(f’ = αdb* * (*t-V*_max —_ *t*_*0*_)^*b*–1^ * exp [– *α* (*t-V*_max_—*t*_*0*_)^*b*^]), and corresponded to the maximum CO_2_ released rate;
- *CO*_2max_ (g.L^–1^) = $\hat{d}$ the total amount of CO_2_ released at the end of the fermentation.

#### Life-history traits (LHT)

During the alcoholic fermentation, cell samples were taken and analyzed as described in the “flow cytometry analysis” session. The experimental measurement of the logarithm of cell concentration was modeled by a discontinuous function of time as described in S2 Supporting Information. The cell size and viability were modeled using a linear model (*W*_*it*_) as described in S3 Supporting Information.

These models allowed computing eight life-history traits (S1 Fig).

- *t-N*_0_ (h) = $\hat{t}N$, the growth lag-phase (time between inoculation and the beginning of population growth);
- *t-N*_max_ (h) = $\hat{t}N_{max}$ the time at which the carrying capacity *K* was reached;
- *r* (logarithm of the number of cell divisions *per* mL *per* hour), the intrinsic growth rate;
- *K* (log[cells/mL]), was the carrying capacity computed as: *K* = *I* + *r* (*t-N*_max_ – *t-lag*) + *C*.
- *J*_max_ (g.h^–1^.10^–8^ cell^–1^) was the maximum value of the estimated CO_2_ production rate divided by the estimated cell concentration.
- *Size-t-Nmax* (μm), the average cell size at *t-N*_max_
- *Viability*.*t-N*_max_ (%), the percentage of living cells at *t-N*_max_
- *Viability*.*t-75* (%), the percentage of living cells at *t-75*.

#### Basic Enological Parameters (BEP)

At the end of the fermentation, six Basic Enological Parameters (BEP) were quantified: *Residual Sugar* (g.L^–1^), *Ethanol* (%vol), *Sugar/Ethanol Yield* (g.L^–1^.%vol^–1^) (ratio between the amount of metabolized sugar and the amount of released ethanol), *Acetic acid* (g.L^–1^ H_2_SO_4_), *Total SO*_*2*_ and *Free SO*_*2*_ (mg.L^–1^). *Residual Sugar* and *Ethanol* were measured by infrared reflectance using an Infra-Analyzer 450 (Technicon, Plaisir, France). For some strains, *Residual Sugar* was below the threshold of detection. In these cases, instead of inferring the value “0”, which is not biologically realistic, we used the value: (*x*/1.05) + *y*, where *x* is the lowest value measured in the whole data and *y* is a value drawn in a uniform distribution ~ U(0, 0.001). *Acetic acid* was quantified by colorimetry (*A*460) in continuous flux (Sanimat, Montauban, France). *Total SO*_*2*_ and *Free SO*_*2*_ were assayed by Pararosaniline titration [71].

#### Aromatic Traits (AT)

The aromatic profile of fermenting yeast was estimated by quantifying 14 aromatic traits (AT). The main volatile compounds were measured at the end of the alcoholic fermentation by GC-MS. For esters, higher alcohols and volatile acids, HSSE HeadSpace Sorptive Extraction followed by GC-MS analysis was used according to Weldegergis *et al*. [72]. For volatile thiols, a specific extraction was performed according to Tominaga *et al*.[73]. These analytical methods allowed us to detect up to 22 compounds in the analyzed samples (S2 Table). However only 13 of them were quantified in a sufficient number of samples and were retained after statistical analysis. These compounds were: two higher alcohols (*Phenyl-2-ethanol* and *Hexanol*, mg.L^–1^), seven esters (*Phenyl-2-ethanol acetate*, *Isoamyl acetate*, *Ethyl-propanoate*, *Ethyl-butanoate*, *Ethyl-hexanoate*, *Ethyl-octanoate* and *Ethyl-decanoate*, mg.L^–1^), three medium chain fatty acids (*Hexanoic acid*, *Octanoic acid* and *Decanoic acid*, mg.L^–1^) and one volatile thiol (*4-methyl-4-mercaptopentan-2-one*, or *4MMP*, ng.L^–1^). For *Ethyl-decanoate* and *Ethyl-octanoate*, which were sometimes below the threshold of detection, we proceeded as described above for *Residual Sugar*. Finally the *Acetate ratio*, ratio between *Phenyl-2-ethanol acetate* and *Phenyl-2-ethanol*, was computed. This parameter represents the acetylation ratio of higher alcohols.

### Data analyses

#### Single-trait analyses

For each of the 35 traits collected, the effects of the strain, of the temperature and of the strain-by-temperature interaction, as well as the random block effect, were estimated through the following mixed model of analysis of variance (R program, *lme4* package):

$$Y_{ijk}=m+si+temp_{j}+{\left(s*temp\right)}_{ij}+W_{k}+E_{ijk}$$

where *Y*_*ijk*_ was the value of the trait for strain *i* (*i* = 1, …, 66) at temperature *j* (*j* = 1, 2) obtained the week *k* (*k* = 1, …, 22), *m* was the overall mean, *s*_*i*_ was the fixed strain effect, *temp*_*j*_ was the fixed temperature effect, (s*temp)_*ij*_ was the interaction effect between temperature and strain, *W*_*k*_ was the random block effect and *E*_*ijk*_ the residual error. For each trait, normality of residual distributions and the homogeneity of the variances were checked. Some of them displayed heteroscedasticity, which decreased the power of the ANOVA. This was due to strains with weak fermentation abilities (*t-lag* > 40 h, *t-V*_max_ > 20 h, *CO*_2max_ < 88 g.L^-1^ and/or *t-75* > 110 h). The predicted means ${\hat{Y}}_{ij}=\hat{m}+\hat{s_{i}}+\hat{t{emp}_{j}}+{\left(\hat{s}\;*\;\hat{temp}\right)}_{ij}$ were computed from the model's parameters, as well as their standard deviations. For many traits a significant block effect was found (α = 0.05). Therefore, the decomposition of the total phenotypic variance of each trait into its genetic and environmental components was computed after correction for the random block effects. Multiple non parametric comparisons (Campbell and Skillings analysis) were carried out using *nparcomp* package of the R program with adjusted *p*-values [74].

### Multi-trait analyses

Principal Component Analysis (PCA) was performed on the ANOVA predicted means for each temperature-strain combination (R program, *ade4* package [75]). The parental strains were added as supplementary individuals. The entire data set used was given in S1 Dataset.

## Results

### Large-scale phenotyping of a half yeast diallel under winemaking conditions

Eleven parental strains (seven strains of *S*. *cerevisiae* and four strains of *S*. *uvarum*, Table 1) and their 55 intra- and inter-specific hybrids were phenotyped under enological conditions, at two temperatures (26°C, favorable for *S*. *cerevisiae*, and 18°C, favorable for *S*. *uvarum*), in three replicates (Fig 1 and S1 Fig). A total of 396 alcoholic fermentations were performed (66 strains * 2 temperatures * 3 replicates). A few fermentations (26) were discarded due to incomplete or absent implantation of the expected strain. The alcoholic fermentations were characterized in depth through 35 phenotypic traits, leading to almost 13 000 numerical data (35 * 370) for all the fermentations. The traits were classified into four categories: (i) Seven fermentation kinetics (FK) parameters: *t-lag*, *t-V*_max_, *t-45t-45*, *t-75*, *AFtime*, *V*_max_, *CO*_2max_; (ii) Eight life-history traits (LHT): *t-N*_0_, *t-N*_max_, *r*, *K*, *J*_max_ (growth traits), *Size t-N*_max_, *Viability t-N*_max_, *Viability t-75* (size and viability traits); (iii) Six basic enological parameters (BEP): *Residual Sugar*, *Ethanol*, *Sugar/Ethanol Yield*, *Acetic acid*, *Total SO2* and *Free SO2*; (iv) Fourteen aromatic traits (AT): *Phenyl-2-ethanol*, *Hexanol*, *Phenyl-2-ethanol acetate*, *Isoamyl acetate*, *Ethyl-propanoate*, *Ethyl-butanoate*, *Ethyl-hexanoate*, *Ethyl-octanoate*, *Ethyl-decanoate*, *Hexanoic acid*, *Octanoic acid*, *Decanoic acid*, *4MMP (4-methyl-4-mercaptopentan-2-one*) and *Acetate ratio*, the acetylation rate of higher alcohols.

### The sources of phenotypic variation differ according to trait categories

The sources of variation of each phenotypic trait were studied by analyses of variance (ANOVA) to estimate the *Strain*, *Temperature*, and Strain*Temperature interaction effects (Table 2). The part of phenotypic variation explained by the model (block effect removed) depended on the trait category, with Fermentation Kinetics parameters (FK) showing the highest *R* ^2^ values (0.60 to 0.92) and Aromatic Traits (AT) the smallest (0.09 to 0.66). All the traits but three (*Isoamyl acetate*, *Ethyl-butanoate* and *Ethyl-octanoate*) displayed a significant *Strain* effect, accounting for 11 to 67% of the variance explained (*p*-value < 0.05). The temperature had contrasted effects according to the trait category: the ten traits for which temperature explained at least 10% of the model variance were mainly found in the Fermentation Kinetics (FK) and Life-history Traits (LHT) categories, with *R* ^2^ values up to 79%: *t-45*, *AFtime*, *V*_max_, *t-75* and *t-lag* (FK), *r*, *J*_max_ and *t-N*_max_ (LHT), *Acetic acid* (BEP), and *Hexanol* (AT). Finally highly significant Strain*Temperature interactions were found for *CO*_*2*max_, *t-lag* and *t-V*_max_ (FK), *t-N*_0_, *K*, *Size*.*t-N*_max_ and *Viability*.*t-75* (LHT), *Ethanol*, *Residual Sugar* and *Sugar/Ethanol Yield* (BEP) and *Acetate ratio* (AT).

**Table 2:** Results of the ANOVAs for 35 variables representative of fermentation and life-history traits in yeast.

Trait	Trait category	Strain number	Mean	Unit	*R* ^2^	Block effect	SS Strain	SS Temp	SS S*T	SS Resid.	*p*-val Strain	*p*-val Temp	*p*-val S*T
*t-lag*	FK	63	19.70	h	0.80	*	0.54	0.23	0.11	0.13	0.00000	0.00000	0.00000
*t-V*_max_	FK	63	8.47	h	0.60	*	0.53	0.06	0.14	0.26	0.00000	0.00000	0.00019
*t-45*	FK	63	31.53	h	0.91	*	0.14	0.75	0.04	0.06	0.00000	0.00000	0.00000
*t-75*	FK	63	71.04	h	0.92	*	0.21	0.68	0.06	0.05	0.00000	0.00000	0.00000
*AFtime*	FK	63	142.39	h	0.91	*	0.20	0.69	0.05	0.06	0.00000	0.00000	0.00000
*V*_max_	FK	63	1.80	g/(L*h)	0.92	ns	0.11	0.79	0.05	0.05	0.00000	0.00000	0.00000
*CO*_*2*max_	FK	63	90.43	g/L	0.65	*	0.45	0.00	0.32	0.23	0.00000	0.07273	0.00000
*t-N*_0_	LHT	63	4.17	h	0.63	*	0.60	0.02	0.15	0.23	0.00000	0.00028	0.00010
*t-N*_max_	LHT	63	28.75	h	0.40	*	0.19	0.32	0.11	0.38	0.02071	0.00000	0.61236
*R*	LHT	63	0.15	log(cells/mL)/h	0.55	*	0.19	0.44	0.08	0.29	0.00040	0.00000	0.66772
*K*	LHT	63	162163078.27	cell/ml	0.36	*	0.32	0.04	0.24	0.40	0.00000	0.00001	0.00025
*J*_max_	LHT	63	0.0047	g/(L*10^8^*cell)	0.40	*	0.36	0.16	0.11	0.38	0.00000	0.00000	0.57488
*Size*.*t-N*_max_	LHT	61	6.13	μm	0.49	*	0.44	0.00	0.26	0.29	0.00000	0.33193	0.00003
*Viability*.*t-N*_max_	LHT	62	90.98	%	0.33	*	0.43	0.00	0.19	0.38	0.00000	0.19832	0.18694
*Viability*.*t-75*	LHT	62	78.20	%	0.64	*	0.50	0.04	0.25	0.20	0.00000	0.00000	0.00000
*Residual Sugar*	BEP	63	1.13	g/L	0.71	*	0.45	0.00	0.35	0.19	0.00000	0.02983	0.00000
*Ethanol*	BEP	63	11.13	%vol	0.68	*	0.55	0.00	0.24	0.21	0.00000	0.66762	0.00000
*Sugar/Ethanol Yield*	BEP	63	16.73	g/L/%	0.50	*	0.50	0.00	0.17	0.32	0.00000	0.76264	0.00041
*Acetic acid*	BEP	63	0.13	g/L	0.38	*	0.33	0.10	0.18	0.40	0.00000	0.00000	0.02117
*Total SO*_2_	BEP	63	172.50	mg/L	0.18	*	0.30	0.02	0.16	0.52	0.00071	0.00923	0.46632
*Free SO2*	BEP	63	67.95	mg/L	0.25	*	0.31	0.00	0.22	0.47	0.00009	0.56268	0.04659
*Phenyl-2-ethanol*	AT	63	191.60	mg/L	0.66	*	0.64	0.02	0.11	0.22	0.00000	0.00000	0.00124
*Hexanol*	AT	63	1.32	mg/L	0.29	*	0.33	0.11	0.10	0.46	0.00000	0.00000	0.91234
*Phenyl-2-ethanol acetate*	AT	63	3.86	mg/L	0.66	*	0.67	0.00	0.11	0.22	0.00000	0.34696	0.00212
*Isoamyl acetate*	AT	63	0.94	mg/L	0.09	*	0.23	0.00	0.18	0.59	0.05512	0.79194	0.54684
*Ethyl-propanoate*	AT	63	0.07	mg/L	0.41	*	0.40	0.06	0.16	0.38	0.00000	0.00000	0.03700
*Ethyl-butanoate*	AT	63	0.05	mg/L	0.11	*	0.20	0.05	0.17	0.58	0.20289	0.00002	0.63904
*Ethyl-hexanoate*	AT	63	0.11	mg/L	0.19	*	0.27	0.08	0.12	0.53	0.00087	0.00000	0.88627
*Ethyl-octanoate*	AT	63	0.06	mg/L	0.11	*	0.23	0.04	0.15	0.58	0.06347	0.00013	0.83217
*Ethyl-decanoate*	AT	63	0.07	mg/L	0.13	*	0.31	0.00	0.13	0.56	0.00042	0.94066	0.89555
*Hexanoic acid*	AT	63	11.16	mg/L	0.20	*	0.33	0.01	0.14	0.52	0.00002	0.02999	0.62936
*Octanoic acid*	AT	63	2.30	mg/L	0.17	*	0.26	0.06	0.14	0.54	0.00518	0.00000	0.87192
*Decanoic acid*	AT	63	0.99	mg/L	0.13	*	0.23	0.04	0.17	0.57	0.03997	0.00015	0.45642
*4MMP*	AT	63	9.28	ng/L	0.44	*	0.51	0.01	0.11	0.36	0.00000	0.00766	0.34848
*Acetate ratio*	AT	63	0.03	-	0.22	*	0.21	0.01	0.27	0.51	0.03548	0.01387	0.00044

*R* ^2^, proportion of variance explained by the model once the block effect has been removed. SS, sum of squares. Temp, temperature. Resid, residual. S*T, strain*temperature interaction. *p*-val, *p*-value.

*, significative block effect at 5%.

Overall, FK and LHT traits displayed *Strain* effects and large *Temperature* effects, and in a lesser extent Strain*temperature interactions (except for *CO*_2max_ with *R* ^2^ = 0.32), BEP traits had both *Strain* and Strain*temperature effects with almost no effect of temperature, and finally AT traits had almost exclusively *Strain* effects.

### Strain types differ for many traits of biotechnological interest

**Fig 2:**
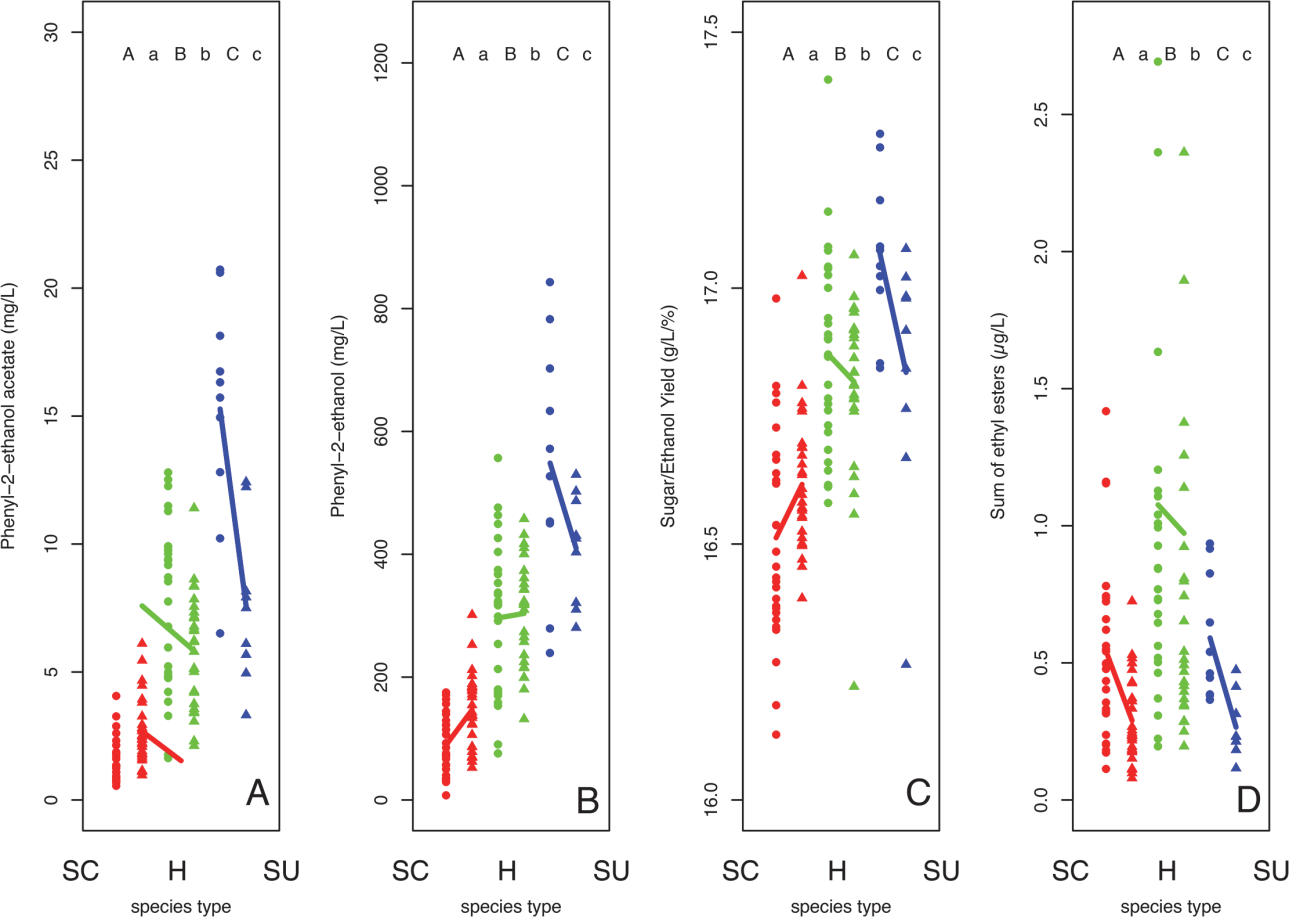
Effect of the hybrid type on some traits of enological interest. *Phenyl-2-ethanol acetate* (A1), *Phenyl-2-ethanol* (A2), *Sugar/Ethanol Yield* (B) and sum of ethyl esters (C) concentrations in *S*. *cerevisiae* (SC), *S*. *uvarum* (SU) and interspecific hybrid (H) strains at 18°C and 26°C. Statistical differences between the species groups were tested for each temperature using a multiple non-parametric test with corrected *p* values (Holm test). Different letters indicate groups showing significant differences (*p* < 0.01). Capital and lower cases were used for 18° and 26°C, respectively.

For each trait*temperature combination, we compared the means of the *S*. *cerevisiae* strains (parents and intraspecific hybrids), of the *S*. *uvarum* strains (parents and intraspecific hybrids) and of the interspecific hybrids, using non-parametric comparison tests (α = 0.05). In 42 cases out of 70 (2 temperatures x 35 traits), at least one mean was significantly different from the others (S2 and S3 Figs). For 12 traits, a difference was observed at both temperatures, for 5 traits at 18°C only and for 12 traits at 26°C only. For some traits of biotechnological interest the three strain types were well separated. As shown Fig 2 (panels A and B), interspecific hybrids had a production of *Phenyl-2-ethanol* and *Phenyl-2-ethanol acetate* that was roughly intermediate between the ones of the parental species. It is well documented that these compounds discriminate *S*. *cerevisiae* and *S*. *uvarum* during wine fermentation. Moreover there was a significant Species*Temperature interaction for these compounds (2% and 6% of variance explained for *Phenyl-2-ethanol* and *Phenyl-2-ethanol acetate*, respectively). Their concentration was significantly lower at 26°C than at 18°C in the *S*. *uvarum* group but this was not the case neither in the *S*. *cerevisiae* group nor in the interspecific hybrids. As a consequence the interspecific hybrids are intermediate between parental species at 18°C and close to the *S*. *uvarum* group at 26°C. Another striking difference between groups was the yield of alcoholic fermentation, a key parameter in winemaking industry because strains with high *Sugar/Ethanol Yield* are required to reduce ethanol content in wine. At 18°C, the *S*. *uvarum* group and the interspecific hybrids required respectively 0.56 and 0.35 g/L more sugar than *S*. *cerevisiae* group for producing 1% vol. of ethanol (Fig 2C). This species discrepancy was highly significant and showed a slight Species*Temperature interaction with a reduced difference between species at 26°C as compared to 18°C. The production levels of several ethyl-esters, was higher in interspecific hybrids than in either parental species at both temperatures. This global heterosis effect was illustrated by summing the concentrations of all ethyl-esters (*Ethyl-propanoate*, *Ethyl-butanoate*, *Ethyl-hexanoate*, *Ethyl-octanoate* and *Ethyl-decanoate*) (Fig 2D). For other traits differences between strain types were found (S2 Fig and S3 Fig). At 26°C the interspecific hybrids produced less acetic acid than the parental species. Finally the production of *4MMP* was significantly lower in the *S*. *cerevisiae* group than in the two other groups. This analysis revealed the existence of large differences between the strain groups analyzed. In addition, the numerous trait*temperature interactions (Table 2) also generated a large phenotypic diversity that may be of interest from a biotechnological viewpoint.

### Intra and Interspecific hybridizations reshape multi-trait phenotypes and improve homeostasis with respect to temperature

**Fig 3:**
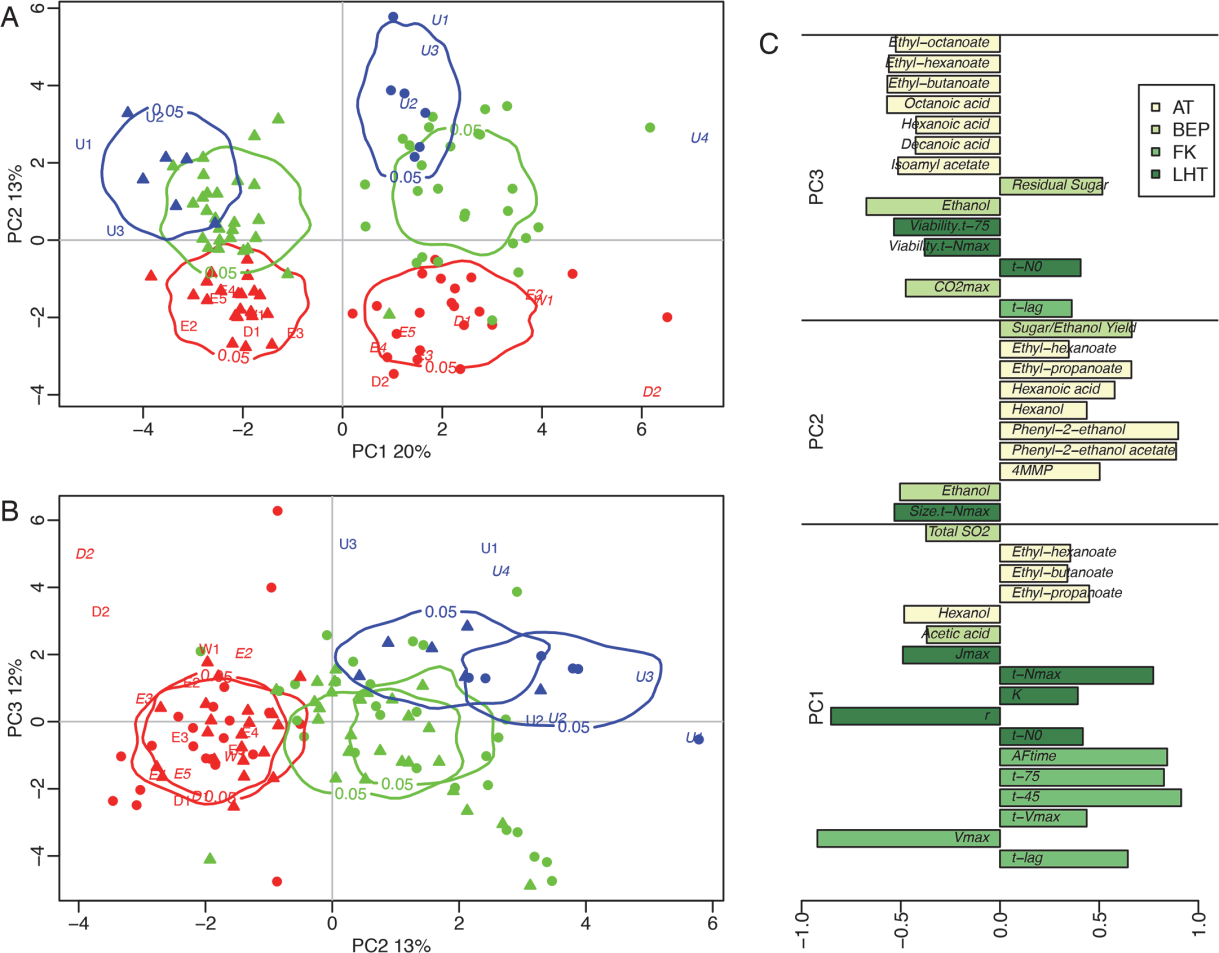
Principal Component Analysis (PCA) performed from the 35 variables listed in Table 2. Each point represents one of the 55 hybrid strains: *S*. *cerevisiae* intraspecific hybrids *S*. *uvarum* intraspecific hybrids and interspecific hybrid at 18°C and 26°C. A: axes 1 and 2 (33% of the total inertia). B: axes 2 and 3 (25% of the total inertia). C: correlation of the variables to discriminant axes PCA1, PCA2 and PCA3. Only variables showing a significant correlation (*p*-value < 0.0001) are shown. The four-color palette corresponds to the four variable categories (FK: Fermentation Kinetics, LHT: Life-history Trait, BEP: Basic Enological Parameters, AT: Aromatic Traits).

In order to capture the consequence of hybridization at the multi-trait phenotypic level, a Principal Component Analysis (PCA) from the whole data set was carried out. The first PCA axis (PCA1) accounted for 20% of the total variance and clearly separated the strains according to the fermentation temperature (Fig 3A). As expected, the first axis was mainly explained by traits showing a large temperature effect in the ANOVA (*p*-value < 0.0001) (Fig 3C). All the FK time traits (*t-lag*, *t-75*, *t-45*, *t-Vmax*, *t-N*_max_) had low values at 26°C, which reduced the alcoholic fermentation time (*AFtime*). These traits were strongly correlated with each other (S4 Fig), which explained the major temperature effect seen on PCA1. The first axis was also clearly explained by *V*_max_ and *r*, two traits with highly significant temperature effects, and in a lesser extent by *K*, *J*_max_, *Acetic acid*, *Hexanol*, *t-N*_0_, *Total SO*_*2*_ and three ethyl esters. The second and the third axes of the PCA (Fig 3B) accounted for 13% and 12% of the total inertia, respectively, and clearly separated the data according to the strain type (*S*. *cerevisiae*, *S*. *uvarum* and interspecific hybrids). Volatile compounds such as *Phenyl-2-ethanol* and *Phenyl-2-ethanol acetate*, as well as most of ethyl esters (AT traits), largely contributed to discriminate the *S*. *uvarum* and *S*. *cerevisiae* groups, which is consistent with their large *R* ^2^ for *Strain* effects. Life-history traits such as cell size and viability also contributed to separate the species. *S*. *uvarum* strains had smaller cell size and a lower viability than *S*. *cerevisiae* strains. Note the negative correlation between carrying capacity *K* and cell size (S4 Fig), previously reported in various studies [65, 76], which were here confirmed in a multi-species context. Finally, three basic enological parameters, *Ethanol*, *Residual Sugar* and *Sugar/Ethanol Yield*, and one FK trait, *CO*_2max_, were also highly correlated to PCA2 or PCA3. Thus both ANOVA and PCA showed that genetic and environmental variations did not affect in the same way the different trait categories. Temperature strongly influenced fermentation kinetics and life-history traits, while fermentation byproducts (AT and BEP) were mainly influenced by strain origin.

**Fig 4:**
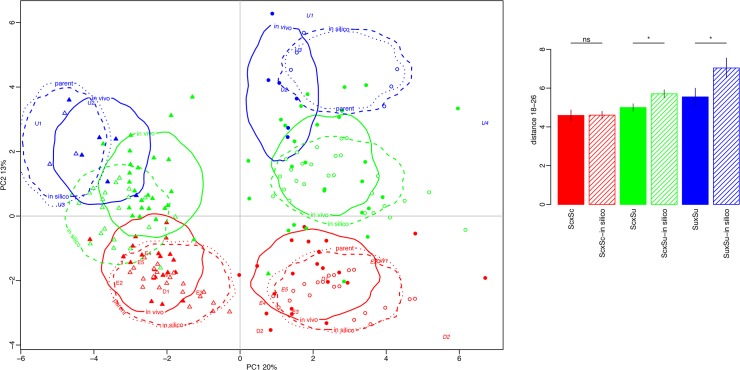
Principal Component Analysis (PCA) of parental strains, *in vivo* and *in silico* hybrids. A. First two dimensions of the PCA, explaining 33% of total variation. The phenotypic values of *in silico* intraspecific and interspecific hybrids were computed assuming additivity (*i*.*e*. mid-parent value) for all 35 traits. B. Multi trait phenotype distance between the two temperatures was measured for each hybrid type. Bar plot represents the mean and the standard error for the six groups of hybrids. Differences between *in silico* and *in vivo* hybrids were tested using a non-parametric (Wilcoxon test).

To assess the relative position of the hybrids and their parents we performed another PCA including *in silico* hybrids. The phenotypic values of *in silico* intraspecific and interspecific hybrids were computed assuming additivity (*i*.*e*. mid-parent value) for all 35 traits. The first discriminating axes accounted for 33% of variance (Fig 4A). As expected, whatever the temperature, the intraspecific *in silico* hybrids perfectly overlapped the groups of their respective parental strains, and the interspecific *in silico* hybrids were intermediary between *S*. *cerevisiae* and *S*. *uvarum* groups. Interestingly, *in vivo* and *in silico* hybrids usually did no overlap very well, meaning that both intra- and interspecific hybridizations created original multi-trait phenotypes that were not mid-way between their parents. The distance between *in silico* and *in vivo* hybrids depended on the hybrid type and on temperature. For example, *in vivo S*. *uvarum* intraspecific hybrids were rather far from their mid-value expectation at both temperatures, as it was the case for interspecific hybrids at 26°C but not at 18°C.

**Fig 5:**
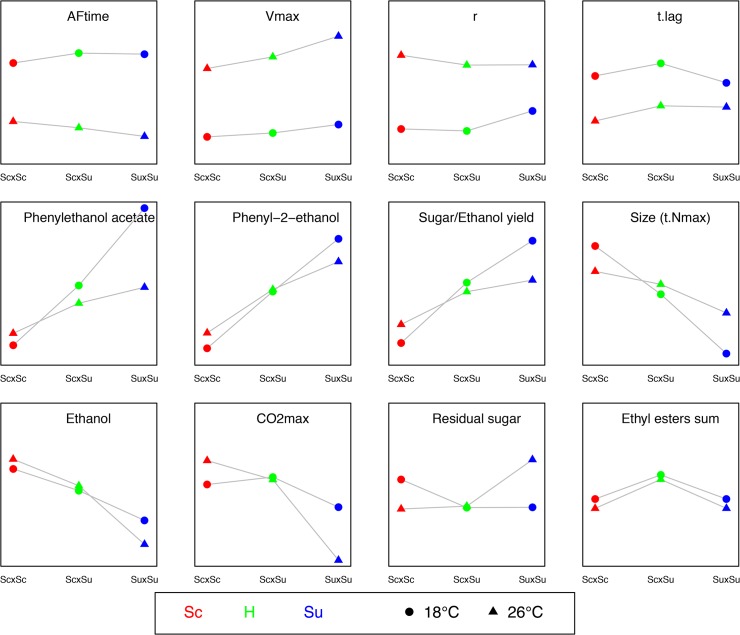
Average values of various traits in the three types of hybrids at 18°C and 26°C. A. Fermentation kinetics traits displaying large *Temperature* effects and moderate *Strain* effects. B and C. Traits displaying *Strain* effects and *Strain* x *Temperature* interactions. Traits in A, B and C are the mainly correlated to axes 1, 2 and 3 of the PCA, respectively. The colors and symbol are the same than previous figures.

For each hybrid type, we computed the multi-trait distance between 18°C and 26°C (Fig 4B). The smaller the distance, the higher the homeostasis with respect to temperature. The *S*. *cerevisiae* hybrids globally showed a higher homeostasis than the others two groups. Moreover homeostasis is significantly higher for *in vivo* hybrids than for *in silico* hybrids for both *S*. *uvarum* and interspecific hybrids. The homeostasis of interspecific hybrids came mainly from *strain* x *temperature* interactions for various traits of biotechnological interest, which display contrasted average values between *S*. *uvarum* hybrids and *S*. *cerevisiae* hybrids (Fig 5). As a result the average values of the interspecific hybrids at 18°C and 26°C were intermediary and close to each other (e.g. *Phenyl-2-ethanol*, *Ethanol*, *Sugar/Ethanol Yield*). For other traits, such as *Residual sugar*, CO_2max_ and sum of ethyl esters, the strict homeostasis observed is not, or not only, due to *strain* x *temperature* interactions (Fig 5).

All these findings indicate that both intra and interspecific hybridization result in multi-trait phenotypes that are hardly predictable from the parental strains and that display, at least for some traits, more homeostasis than expected under the additivity hypothesis.

## Discussion

### Diallel design

In this study, a diallel design of 55 newly synthetized hybrids was obtained from 11 parental strains belonging to the two main species involved in grape juice fermentation, *S*. *cerevisiae* and *S*. *uvarum*. This kind of genetic design has been widely used in plant and animal breeding to analyze the genetic bases of complex traits and identifying heterotic groups [4, 77]. In yeast, a diallel design has been recently developed by different authors using a collection of *S*. *cerevisiae* yeast strains [14–17]. Our design included for the first time interspecific hybrids, allowing us to investigate possible synergies between the genomes of *S*. *cerevisiae* and *S*. *uvarum*. From 370 controlled fermentation experiments at two temperatures in a natural grape juice (Sauvignon blanc), we measured or estimated through mathematical models various fermentation kinetics parameters, life-history traits and a series of metabolites including wine aromatic compounds such as esters and volatile thiols, resulting in about 13 000 data points for 35 phenotypic traits. Global characteristics of intra- and interspecific hybrids were described, focusing in particular on their possible biotechnological interest.

### Interspecific hybridization between *S*. *cerevisiae* and *S*. *uvarum* strains provides yeasts with new and suitable traits for winemaking

Multivariate analysis clearly showed that interspecific hybrids can be separated from *S*. *cerevisiae* and *S*. *uvarum* strains mostly by aromatic traits and other parameters crucial for enology (Fig 3B and 3C). Hybridization between *S*. *cerevisiae* and *S*. *uvarum* strongly reshapes the production of several secondary metabolites in interspecific hybrids (S2 Fig and S3 Fig). This finding was previously reported for glycerol [45], acetic acid [41, 45], volatile thiols (*4MMP*) [46] and higher alcohols such as *Phenyl-2-ethanol* [31, 41, 44]. Except for glycerol that was not assayed here, these observations were confirmed for a large set of hybrids. At 26°C, the interspecific hybrids produced less acetic acid than the parental species, which can be useful for wine yeast selection [78]. Interestingly, at the same temperature, the *4MMP* production was three fold higher in interspecific hybrids and *S*. *uvarum* group than in *S*. *cerevisiae* group. This can be explained by the inheritance of the dominant *Irc7p* allele of *S*. *uvarum* encoding for a fully active a cystathionin β-lyase able to cleave efficiently the cysteinylated precursor of this compound [79, 80]. The production of *Phenyl-2-ethanol* and its acetate in the interspecific hybrids confirmed to be intermediate between the parental species [46]. The high level of these molecules is a major feature of *S*. *uvarum* species and could be due to the more active shikimate and phenylalanine pathways found in this species [54, 81]. Interestingly interspecific hybrids produced lower amount of these compounds than *S*. *uvarum*. In wine these compounds can mask more subtle fragrances [46], so their moderate production during alcoholic fermentation is desired.

Beside these already described features, our data provide new interesting findings. First, interspecific hybrids display a much higher production of ethyl esters (2.45 folds) than parental species at both temperatures. These compounds positively impact wine quality by conferring fruity notes [82]. The production of ethyl esters can be related to two factors: (i) the availability of short and medium fatty acid chains which depends on the must composition, the fermentation temperature [83, 84] and the species [84]. *S*. *uvarum* and *S*. *cerevisiae* show large differences in lipid metabolism [81] with a higher level of Medium Chain Fatty Acids (MCFA) production in *S*. *uvarum* [84], likely due to a more active fatty acid pathway [54]; (ii) the esterification of these fatty acids with ethanol that is mediated by specific ethanol-O-Acyl transferases (EOAT) (Eeb1p, Eht1p, Ymr210p) [85, 86]. Recent gene expression surveys demonstrated that allele *Eeb1p* of the major EOAT was much more expressed in *S*. *cerevisiae* than in *S*. *eubayanus* [87], or in *S uvarum and S kudriavzevii* [88]. This could indirectly suggest that *S*. *cerevisiae* might have a higher EOAT than *S*. *uvarum*, a species closely related to *S*. *eubayanus* [89]. The high production of ethyl esters in interspecific hybrids could therefore result from the combination of these two factors. A second interesting result was the higher *Sugar/Ethanol Yield* found in both interspecific hybrids and *S*. *uvarum* strains as compared to *S*. *cerevisiae* strains. To date the natural intraspecific variation among *S*. *cerevisiae* strains was very low for this trait [47, 65, 66]. Due to the continuous increasing level of ethanol in wines, the Sugar/Ethanol Yield is becoming an important trait for wine yeast selection [90–92]. Recent works demonstrated that *S*. *uvarum* and *S*. *kudriavzevii* species have a Sugar/Ethanol Yield higher than *S*. *cerevisiae*, especially at low temperature [29, 41]. However these species are susceptible to high ethanol content and elevated temperature and are not adapted to harsh fermentation conditions. Additional investigations with higher sugar concentrations confirmed that some of these hybrids can reduce the ethanol content in wine up to 0.4% without excessive production of acetic acid [93].

The collection of 28 interspecific hybrids obtained also allows the investigation of mitochondrial inheritance effect for the 35 traits. Using the multi locus (*ATP6*, *COX2*, *COX3*) molecular typing described by Albertin *et al*. [62] we determined the mitochondrial inheritance of these hybrids (10 mt-Sc, 17 mt-su, 1 not determined). However we failed to establish a statistical link between this mitochondrial inheritance and trait variation (data not shown). This result suggested that the mitochondrial inheritance has a small impact during the alcoholic fermentation. This conclusion has been previously reported using isogenic interspecific hydrids harboring different mitochondrial inheritance in two previous studies [62, 94].

### Hybridization results in homeostasis for some traits and creates phenotypic novelties

Temperature had a major effect on many variables, particularly on the fermentation kinetics traits, and numerous *strain* x *temperature* interactions could be detected (Fig 5 and Table 2). From a multivariate analysis including both *in vivo* and *in silico* hybrids, we showed that the *in vivo S*. *cerevisiae* hybrids are more homeostatic than interspecific and *S*. *uvarum* hybrids, but it is worth noting that hybridization has the largest effect on homeostasis in *S*. *uvarum*. In the interspecific hybrids, homeostasis was observed for traits of biotechnological interest such sugar/ethanol yield and aroma production (Figs 2 and 5).

Beside homeostasis, both intra- and interspecific hybridization were shown to create novel multi-trait phenotypes. The occurrence of such an effect of hybridization was previously occasionally described, essentially for plant inter-specific hybrids of *Brassica* sp., *Gossypium* sp., etc. [95]. In addition, the extent of phenotypic novelty depends on temperature, suggesting that environmental conditions may modulate the phenotypic innovation associated with intra- and inter-specific hybridization. Our results show that both intra- and inter-specific hybridization can generate hybrids departing from their parents. For example, interspecific hybrids performed better than their parents for ethyl esters production. Such phenotypic transgression, associated with homeostasis, is particularly interesting from an evolutionary viewpoint. Interspecific hybrids with robust fitness are more likely to colonize winemaking environments that are basically changing. Alternatively, homeostasis for Basic Enological Parameters, Fermentation Kinetics and Aromatic Traits may have been selected by human for winemaking, allowing the dissemination of strains having quite stable phenotypes over temperature changes. Conscious or unconscious anthropic selection may explain why intra- and inter-specific hybridization is so frequent in yeast. Indeed, numerous natural hybrids were described associated with enology [20, 30, 33], but also with other bioprocesses producing alcoholic beverages (beer, cider, etc.) [41, 89, 96]. Altogether, homeostasis, phenotypic novelties and transgressive phenotypes may explain the evolutionary role of hybridization in natural or domesticated yeasts.

## Supporting Information

S1 Dataset (CSV)

S1 Fig Fermentation kinetics and population dynamics parameters during alcoholic fermentation. Panel A. Fermentation kinetics: CO_2_ released was expressed in g.L^–1^; *t-lag* (h) corresponded to the time between inoculation and the beginning of CO_2_ release; *t-45* (h) and *t-75* (h) were respectively the fermentation time at which 45 g.L^-1^ and 75 g.L^-1^ of CO_2_ were released, excluding *t-lag*; *AFtime* (h) was the time necessary to ferment all the sugars in the medium excluding *t-lag*, and *CO*_*2*max_ (g.L^–1^) corresponded to the total amount of CO_2_ released at the end of the fermentation. Panel B. CO_2_ production rate was expressed in g.L^–1^.h^–1^; *V*_max_ (g.L^–1^.h^-1^) corresponded to the maximum CO_2_ production rate; *t-V*_max_ (h) was the fermentation time at which *V*_max_ was reached. Panel C. Cell growth: the carrying capacity *K* was expressed in cell.mL^–1^; *t-N*_*0*_ (h) and *t-N*_*max*_ (h) were respectively the time to reach the initial growth point and the carrying capacity *K*. Panel D. CO_2_ flux, *J*, computed by dividing the CO_2_ production rate by the estimated cell concentration (g.h^–1^.10^8^ cell^–1^). *J*_max_ is the maximum flux. Panel E. Evolution of cell *Size* (diameter, μm) over time. *Size-t-Nmax* (μm) was the average cell size at *t-N*_max_. Panel F. Evolution of *Viability* over time. *Viability*.*t-N*_max_ and *Viability*.*t-75* (%) were the percentages of living cells at *t-N*_max_ and *t-75*, respectively.
(PDF)

S2 Fig Traits with a significant species effect at 18°C. (PDF)

S3 Fig Traits with a significant species effect at 26°C. (PDF)

S4 Fig Correlation between the 35 fermentation traits analyzed at 18°C (A) and 26°C (B). Only parameters showing a significant correlation (p-value < 0.05 after Benjamini-Hochberg adjustment) were represented by a dot. Green and red tones correspond to positive and negative correlation, respectively.
(PDF)

S1 Supporting Information CO_2_ production measurement. (PDF)

S2 Supporting Information Cell growth measurement. (PDF)

S3 Supporting Information Cell size and viability traits. (PDF)

S1 Table List of primers used for microsatellite analysis. (PDF)

S2 Table List of compounds measured and analyzed. (PDF)
