# Supplementary figures and images for: Hybridization within Saccharomyces Genus Results in Homoeostasis and Phenotypic Novelty in Winemaking Conditions
Source: PLoS One. 2015 May 6;10(5):e0123834. doi: 10.1371/journal.pone.0123834 (PMC4422614; doi:10.1371/journal.pone.0123834)

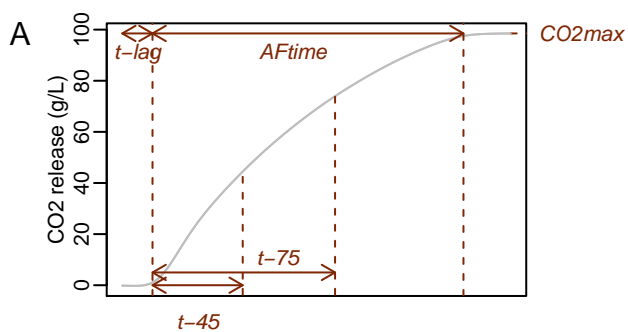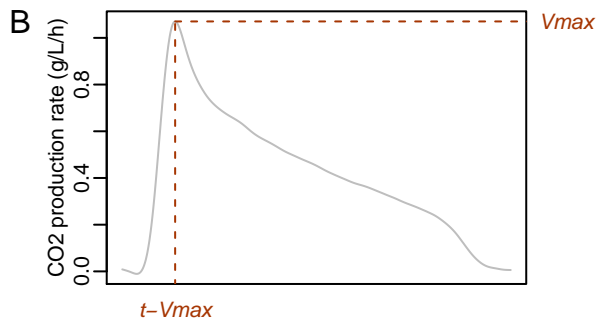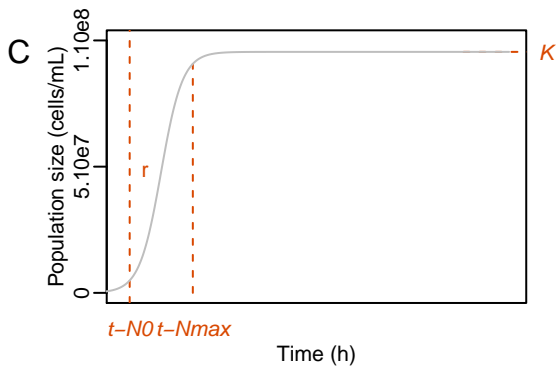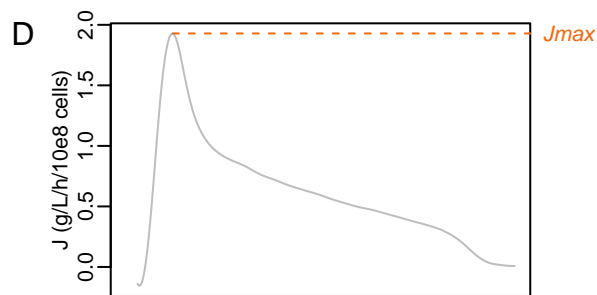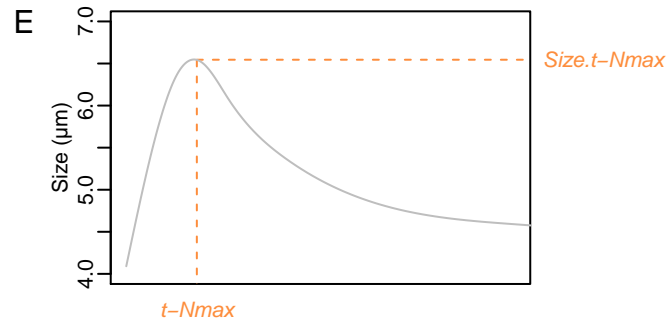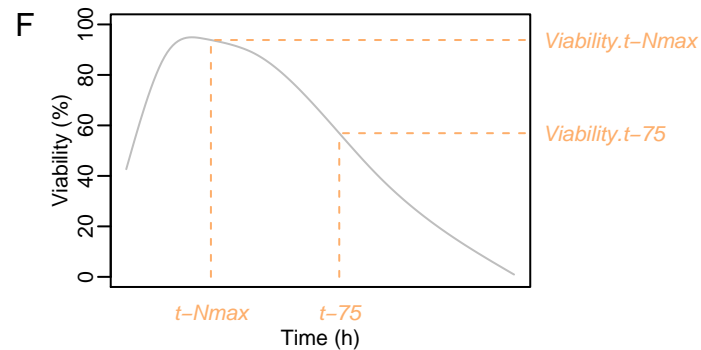

Supplement: S1 Fig — Panel A. Fermentation kinetics: CO2 released was expressed in g.L–1; t-lag (h) corresponded to the time between inoculation and the beginning of CO2 release; t-45 (h) and t-75 (h) were respectively the fermentation time at which 45 g.L-1 and 75 g.L-1 of CO2 were released, excluding t-lag; AFtime (h) was the time necessary to ferment all the sugars in the medium excluding t-lag, and CO 2max (g.L–1) corresponded to the total amount of CO2 released at the end of the fermentation. Panel B. CO2 production rate was expressed in g.L–1.h–1; V max (g.L–1.h-1) corresponded to the maximum CO2 production rate; t-V max (h) was the fermentation time at which V max was reached. Panel C. Cell growth: the carrying capacity K was expressed in cell.mL–1; t-N 0 (h) and t-N max (h) were respectively the time to reach the initial growth point and the carrying capacity K. Panel D. CO2 flux, J, computed by dividing the CO2 production rate by the estimated cell concentration (g.h–1.108 cell–1). J max is the maximum flux. Panel E. Evolution of cell Size (diameter, μm) over time. Size-t-Nmax (μm) was the average cell size at t-N max. Panel F. Evolution of Viability over time. Viability.t-N max and Viability.t-75 (%) were the percentages of living cells at t-N max and t-75, respectively. (PDF) [file pone.0123834.s002.pdf]

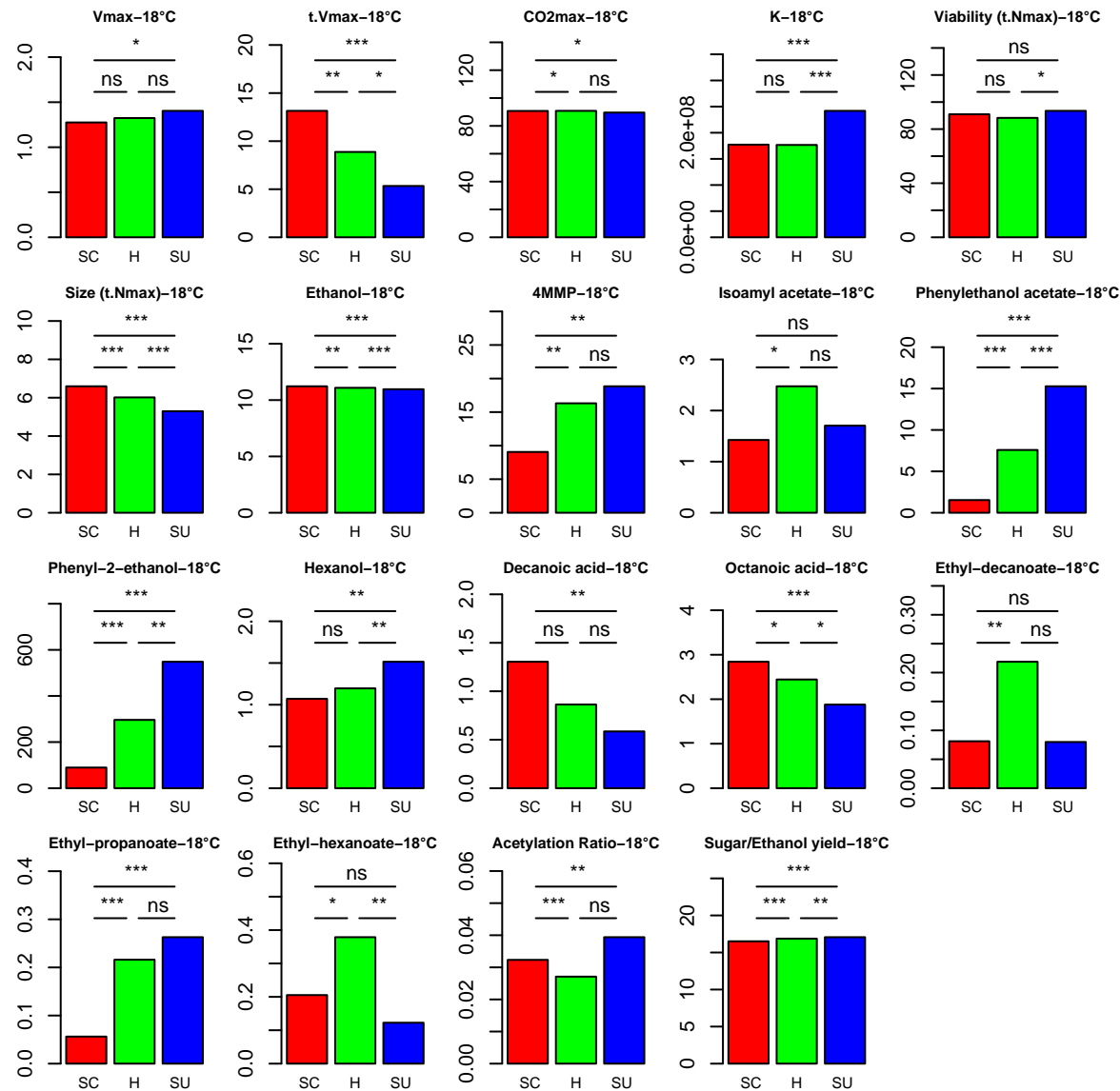

Supplement: S2 Fig — (PDF) [file pone.0123834.s003.pdf]

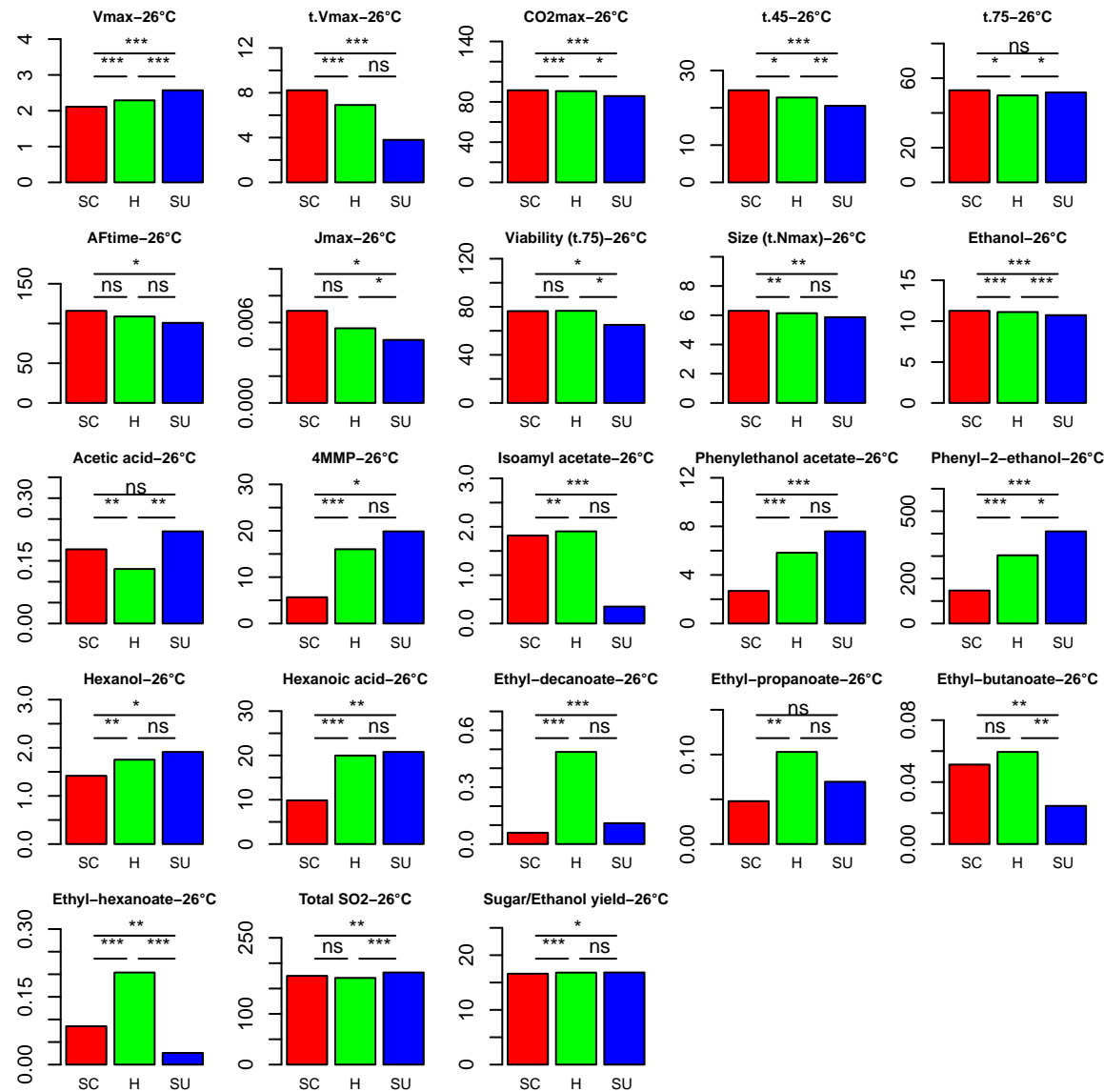

Supplement: S3 Fig — (PDF) [file pone.0123834.s004.pdf]

fermentations at 18°C

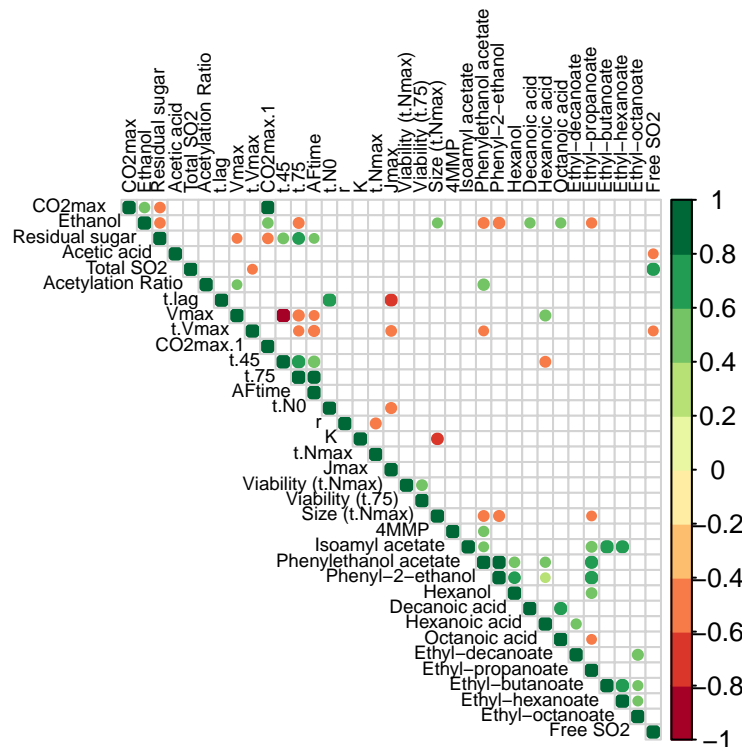

fermentations at 26°C

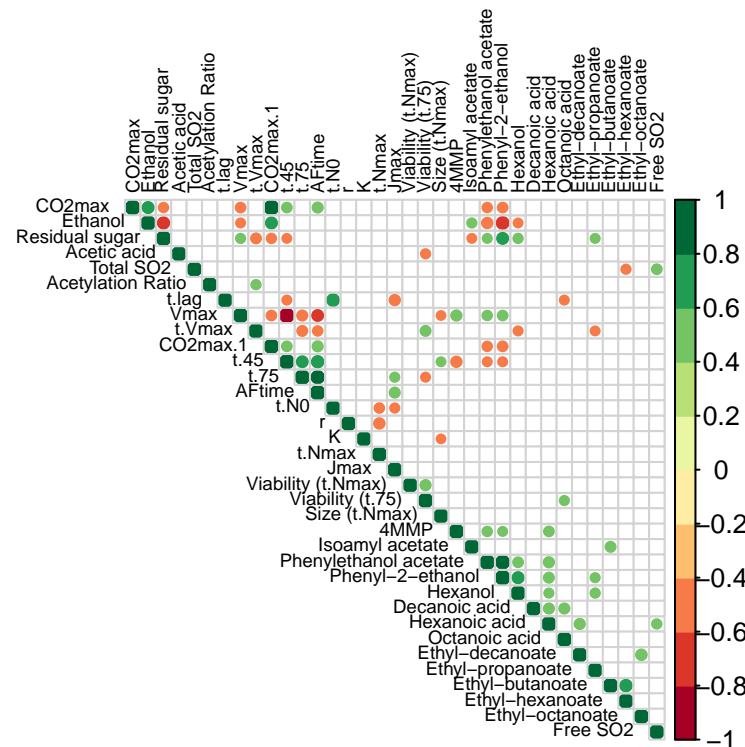

Supplement: S4 Fig — Only parameters showing a significant correlation (p-value < 0.05 after Benjamini-Hochberg adjustment) were represented by a dot. Green and red tones correspond to positive and negative correlation, respectively. (PDF) [file pone.0123834.s005.pdf]
