# Supplementary material for: Hybridization within Saccharomyces Genus Results in Homoeostasis and Phenotypic Novelty in Winemaking Conditions
Source: PLoS One. 2015 May 6;10(5):e0123834. doi: 10.1371/journal.pone.0123834 (PMC4422614; doi:10.1371/journal.pone.0123834)
Supplement: S1 Supporting Information — (PDF) [file pone.0123834.s006.pdf]

S1_Supporting information

The amount of CO_2 released (Y_{it}) for the fermentation i at time t was modeled as

$$Y_{it} = f\,(t, t_{0i}, d_i, \alpha_i, b_i) + \varepsilon_{it}$$

with [ε_{it}] i.i.d. ~ N (0, $\sigma_{f,i}^{2}$). Function f was a discontinuous function of time t, allowing for a lag-time of duration t_0. The lag-time corresponds to the gradual saturation of the medium by carbon dioxide, implicating the CO_2 released was undetectable before it. After t_0, the release of CO_2 was supposed to follow a Weibull function of parameters (d, α, b) (Figure 2A and Figure S1):

$$\begin{cases} t \le t_0: \; f\,(t) = 0 \\ t \ge t_0: \; f\,(t, t_0, d, \alpha, b) = d\,(1 - \exp[-\,\alpha\,(t - t_0)^{b}]\,) \end{cases}$$

where d was the total amount of CO_2 released at the end of the fermentation (*t.end*), α was a shape parameter and b was a parameter that gave information on the presence ($b > 1$), or absence ($b < 1$) of an inflection point in the curve (Figure S1). Based on common fermentation knowledge, we applied two additional constraints to the model: $d \le 93$ g.L^{-1} and $b > 1$.

A grid of values was first assigned to t_{0i}. For each t_{0i} value, the parameters d_i, a_i and b_i were estimated from nonlinear least squares, through an iterative procedure by using nls2 R package (Huet 1996). At the end, the set of four parameters $\left(\hat{t}_{0i}, \hat{d}_i, \hat{a}_i, \hat{b}_i\right)$ minimizing the residual sum of squares between Y_{it} and $f\left(t, \hat{t}_{0i}, \hat{d}_i, \hat{a}_i, \hat{b}_i\right)$ was selected. The homogeneity and independence of the residues was checked by pooling all fermentations and plotting the residues against the fitted values (Figure S2). The standardized residuals ranged between – 3 and + 3 indicating that the model fitted well the data. The structure found for the residuals indicated that the amount of CO_2 computed from the model underestimated or overestimated the amount of CO_2 released, depending on the progress of the fermentation. A better fit could have been obtained at the price of an increase of the number of parameters. Since the number of data points was not large enough, we chose to keep the Weibull model for the CO_2 release after the lag time. Because all strains were treated in the same way, this did not impair the conclusions of the work.
