# Supplementary material for: Hybridization within Saccharomyces Genus Results in Homoeostasis and Phenotypic Novelty in Winemaking Conditions
Source: PLoS One. 2015 May 6;10(5):e0123834. doi: 10.1371/journal.pone.0123834 (PMC4422614; doi:10.1371/journal.pone.0123834)
Supplement: S2 Supporting Information — (PDF) [file pone.0123834.s007.pdf]

# S2_Supporting information

Experimental measurements of the logarithm of cell concentration ($Z_{it}$) for each alcoholic fermentation $i$ at time $t$, was modeled as: $Z_{it} = g\ (t, t_{Ni}, t_{N\mathrm{max}i}, r_i, m_i, I_i, C_i) + \varepsilon_{it}$, with $[\varepsilon_{it}]$ i.i.d. ~ N $(0, \sigma_{g,i}^{2})$ and where $g$ was a discontinuous function of time. We assumed that after a lag-time of duration $t_N$, each cell population grew exponentially and reached its carrying capacity at time $t_{N\mathrm{max}}$. Then, the population size could either stay constant (reduced model R) or change exponentially at a different rate due to mortality (full model F). Under model F, the function $g$ writes:

$$
\begin{cases}
t \leq tN: & g\ (t, tN, tN_{\mathrm{max}}, r, m, I, C) = I \\
tN < t < tN_{\mathrm{max}}: & g\ (t, tN, tN_{\mathrm{max}}, r, m, I, C) = I + r\ (t - tN) \\
t \geq tN_{\mathrm{max}}: & g\ (t, tN, tN_{\mathrm{max}}, r, m, I, C) = I + r\ (tN_{\mathrm{max}} - tN) + C + m\ (t - tN_{\mathrm{max}})
\end{cases}
$$

where $tN$ (h) was the lag-time, $tN_{\mathrm{max}}$ (h) was the time to reach the carrying capacity, $I$ (log[cells/mL]) was the initial cell concentration, $r$ (logarithm of the number of cell divisions *per* hour) was the growth rate, $m$ was the growth rate after $tN_{\mathrm{max}}$, and $C$ (log[cells/mL]) was a parameter which accounted for the possible lack of experimental points around $tN_{\mathrm{max}}$.

For each model, a grid of values was first assigned to $tN_i$ (model R) or to the couple ($tN_i$, $tN_{\mathrm{max}i}$) (model F). For each model and each possible values, parameters $r_i$, $m_i$, $I_i$ and $C_i$, were obtained from segmented linear regression using lm and home-written code in R-software. The different models were then compared using the Akaike Information Criterion (AIC) and the best model was chosen. Notice that under model R, $m_i = C_i = 0$ and $t.N_{\mathrm{max}}$ varied between $tN$ and 378 h which corresponded to the latest experimental time point among all realized fermentations. The homogeneity and independence of the residuals were checked by pooling all fermentations and plotting the residuals against the fitted values.
