# Supplementary material for: Hybridization within Saccharomyces Genus Results in Homoeostasis and Phenotypic Novelty in Winemaking Conditions
Source: PLoS One. 2015 May 6;10(5):e0123834. doi: 10.1371/journal.pone.0123834 (PMC4422614; doi:10.1371/journal.pone.0123834)
Supplement: S3 Supporting Information — (PDF) [file pone.0123834.s008.pdf]

S3_Supporting information

Experimental measurements of cell size (W_{it}) for each alcoholic fermentation i at time t were assumed to follow a linear model: $W_{it} = H + w.t + \varepsilon_{it}$, $[\varepsilon_{it}]$ i.i.d. $\sim$ N $(0, \sigma_{w,i}^2)$ between $t.N_{\max}$ and the time at which 93 % of the total amount of CO_2 was released. All the data points outside this time range were discarded for the following reasons: (i) cell size tended to increase before $t.N_{\max}$ but we did not have enough experimental data points before $tN_{\max}$ to fit such a model; (i) cells tended to flocculate at the end of the fermentation and we observed that the data were no more reliable beyond about 93 % of total amount of CO_2 released. Again, after fitting the model, the homogeneity and independence of the residuals was checked by pooling all fermentations and plotting the residuals against the fitted values.
