# Supplementary material for: Hybridization within Saccharomyces Genus Results in Homoeostasis and Phenotypic Novelty in Winemaking Conditions
Source: PLoS One. 2015 May 6;10(5):e0123834. doi: 10.1371/journal.pone.0123834 (PMC4422614; doi:10.1371/journal.pone.0123834)
Supplement: S1 Table — (PDF) [file pone.0123834.s009.pdf]

S1_Table. List of primers used for microsatellite analysis.

name	labbel	locus	sequence	final concentration µM	reference
pC5fw	Fam	*YFR038*	gtgtcttgacacaatagcaatggccttca	0.3	[31]
pC5rev			gcaagcgactagaacaacaatcaca	0.3	
p91fw	Ned	*YML091*	gtgtctaagcctcttcaagcatgac	1.0	
p91rev			ctgtctggacaattttgccacctta	1.0	
p703	Fam	Locus 4	ggacactagagttcgtctcg	0.3	[32]
p704			gccaccactatcagttcg	0.3	
p705	Ned	Locus 9	cacggcaatcagcacattt	1.0	
p706			tgaagtttcatcatcggcaa	1.0	
