# Supplementary material for: Hybridization within Saccharomyces Genus Results in Homoeostasis and Phenotypic Novelty in Winemaking Conditions
Source: PLoS One. 2015 May 6;10(5):e0123834. doi: 10.1371/journal.pone.0123834 (PMC4422614; doi:10.1371/journal.pone.0123834)
Supplement: S2 Table — (PDF) [file pone.0123834.s010.pdf]

# S2_Table. List of compounds measured and analyzed.

| class | unit | compound | retained |
|---|---|---|---|
| esters | mg/L | *Hexyl acetate* | *no* |
| esters | mg/L | *Isoamyl acetate* | *yes* |
| esters | mg/L | *2-Phenylethanol-acetate* | *yes* |
| esters | mg/L | *Ethyl 2-Methylpropanoate [mC3C2]* | *no* |
| esters | mg/L | *Ethyl butanoate [C4C2]* | *yes* |
| esters | mg/L | *Ethyl 2-Methylbutanoate [mC4C2]* | *no* |
| esters | mg/L | *Ethyl hexanoate [HC6C2]* | *yes* |
| esters | mg/L | *Ethyl propanoate [C6C2]* | *yes* |
| esters | mg/L | *Ethyl octanoate [C8C2]* | *yes* |
| esters | mg/L | *Ethyl decanoate  [C10C2]* | *yes* |
| fatty acid | mg/L | *Butyric acid* | *no* |
| fatty acid | mg/L | *Isobutyric acid* | *no* |
| fatty acid | mg/L | *Propionic acid* | *no* |
| fatty acid | mg/L | *Isovaleric acid* | *no* |
| fatty acid | mg/L | *Hexanoic acid* | *yes* |
| fatty acid | mg/L | *Octanoic acid* | *yes* |
| fatty acid | mg/L | *Decanoic acid* | *yes* |
| higher alcohols | mg/L | *Hexanol* | *yes* |
| higher alcohols | mg/L | *2-Phenylethanol* | *yes* |
| volatile thiols | ng/L | *3-mercapto-hexan-1-ol* | *no* |
| volatile thiols | ng/L | *4-methyl-4-mercaptopentan-2-one* | *yes* |
| volatile thiols | ng/L | *3-mercapto-hexyl acetate* | *no* |
